# Implementing integrated care in ophthalmology: a community case study

**DOI:** 10.3389/fmed.2023.1150525

**Published:** 2023-12-27

**Authors:** Eleni Nikita, Richard Allen, Hussein Khambati, Christos Tsounis, William Tucker, Kirsten Malcolmson, Melanie Hingorani, Zoe Marjoram

**Affiliations:** Moorfields Eye Hospital NHS Foundation Trust, London, United Kingdom

**Keywords:** block contract, implementation, integrated care, ophthalmology, optometry

## Abstract

**Introduction:**

In 2017, in a context of financial and patient care challenges, Moorfields Eye Hospital in the borough of Croydon launched the first Ophthalmology Integrated Care Contract in the United Kingdom.

**Description:**

A realistic, systematic approach is presented for an efficient implementation of an integrated care ophthalmology contract under a lead provider. The main elements of the new contract are portrayed.

**Discussion:**

A new healthcare contract that would lead to system-wide transformation requires significant time commitment, vision, shared narrative, leadership, multi-functional working culture, shared accountability of all participating parties and education and support of all parties involved. Key levers to elevate the quality of care are collaborative relationships between health professionals, investing in information and technology and facilitating bottom-up innovation.

**Conclusion:**

System-wide changes such, as integrated care contracts are possible, although the interplay between context, design and implementation is more complex than expected.

## Introduction

For nearly a decade, the National Health System (NHS) in the United Kingdom (UK) has experienced a significant mismatch between growth of funding and growth in demand, and delivery costs, for services. In 2019 the NHS marked its 70th anniversary by publishing the *NHS long-term plan*, (1) building on the policy platform laid out in the *NHS five-year forward view*, (2) to address these challenges through integrating care, technology and innovation, and workforce.

Ophthalmology is the largest outpatient service in the UK NHS and is recognized as a key policy area for transformation, incorporating pathways which integrate primary and secondary eye care (3, 4). However, the implementation of these integrated services consistently at scale is slow, with numerous systems struggling to overcome challenges including clinical governance, contracting and flow of information. This paper presents a realistic and systematic approach to implementation of an ophthalmology integrated care contract using a lead provider approach. Our purpose is to demonstrate that this is feasible even with current constraints, and extract several transferable lessons for other regions and countries.

### The local setting

Croydon is the second largest borough in London, with a very ethnically diverse population of 386,000, among which 15% fall into the highest category of deprivation (5). Moorfields Eye Hospital (MEH) had been delivering a comprehensive subspecialty-based secondary, tertiary and emergency ophthalmology service for Croydon since 2014, mainly from Croydon University Hospital, but also from smaller community clinics, with access to the main central London site when required. This was undertaken under a payment by results contract with NHS Croydon Clinical Commissioning Group (CCG), with MEH undertaking 95% of the ophthalmology work for the CCG’s population. In the UK, CCGs are responsible for about 60% of the NHS budget and commission most secondary and primary care services (6).

The annual pre-pandemic activity (year 2018/2019) was 53 K outpatient attendances and 2.6 K elective procedures.

In parallel, since 2012, a cluster of 14 primary care optometry practices collaborated under a community optometric umbrella organization, to offer enhanced primary care ophthalmology services contracted directly by the CCG. These services included minor eye care conditions service (MECS) and referral filtering for cataract and glaucoma. Support to the local visually impaired population was provided separately by an independent ophthalmic charity.

In October 2017, the CCG served notice on the existing services, as they became financially unsustainable in the face of increasing demand. The CCG sought to develop a service, under a Lead Provider structure, to integrate the specialist hospital and community-based eye care, and reduce the volume of patients seen in the hospital, via a shift to management within the community whilst improving overall performance, quality and patient experience.

## Description of the care practice

### Materializing a ‘first of a kind’ contract

From early 2018, an Integrated Ophthalmology Steering Group was formed to oversee clinical work streams, which had representation from primary, community, secondary and third sector providers, as well as a patient reference group. The steering group agreed final clinical pathways in December 2018 (14 months post initial service termination notice), which allowed the CCG to develop the service specification and business case, approved in partnership with all stakeholders in April 2019. The CCG then required that MEH and the community optometry provider (COP) successfully completed a full tender assurance process, including confirmation on operational readiness, regulatory compliance, governance structures and safeguarding.

The mobilization phase of the new integrated care contract was led by MEH and commenced in July 2019. The CCG had a very tight timeline of only 3 months to a go-live and the MEH Board allocated a formal project manager to the task. A detailed mobilization plan was established, defining stakeholders, working groups, meeting frequency per group, membership and key milestones, as well as a detailed risk register with mitigation plans (see Supplementary Table 1). The redesigned emergency services were able to go live as soon as the business case was approved; given the short time frame, the commissioners and Moorfields agreed the other key service specification elements which needed to be in place for the go-live date: the MEH contract variation, the COP sub-contract for pre-existing pathways and a ‘Single Point of Access for referrals and triaging within the Croydon borough.

### How does the contract work?

The financial arrangement uses two block contracts. The first is between MEH and the CCG, with MEH responsible for ensuring the delivery of all aspects of the service. The second is between MEH and the COP, and the latter has to subcontract a range of accredited optometry practices/optometrists to deliver the service for the community-based care. The quality standards of care were determined by MEH and agreed by all sides.

To develop the business case and consequent contracting between MEH and the CCG, and between MEH and the COP, a number of assumptions were used, based on the existing activity, with adjustments for likely local demand growth, the level of projected efficiencies and the degree of shift of patient care into the community optometry settings per subspecialty. The latter aspects were informed by local demographic information, published evidence on incidence/prevalence of disease in populations, audits of patient diagnoses and outcomes of clinical visits which identified the level of cases suitable for potential community care, and audits of the quality of referrals including the level of inappropriate and avoidable referrals.

The block contract has a “cap and collar” approach: there is a block financial envelope, with a 2% risk share where, if activity increases or decreases beyond 2%, the contract moves to a cost & volume arrangement, which is capped at 5%. The block contract between MEH and the COP is based on the likely activity combined with the average tariff used for existing community services in other parts of the country; for example, the MECS tariff was based on agreed cost of average £64 per attendance at COP (new or follow up). The only element outside of this block contract is assessment of postoperative cataract patients, which is on a cost & volume basis as the service was starting from scratch, at £50 per attendance.

Progress is monitored at a monthly Contracts, Performance and Quality meetings with the CCG, to assess performance against the agreed performance KPIs, and to monitor the assumptions underpinning the contract. This is critical in identifying variations early so relevant investigations can take place to understand the cause, impact on the financial contract and to consequently take action if necessary.

### The elements of the integrated care contract

The elements of the Integrated Care service are the following.

#### Single point of access (SPA) for all routine referrals

This is a single electronic referral route for ophthalmology, via a one-click standardized referral form on NHS e-Referral Service (e-RS) (7) for the GPs and COP optometrists. Non-COP optometrists send a standardized referral form (General Ophthalmic Services 18 [GOS18]) to COP optometrists via a secure nhs.net mail (8). A team of COP optometrists triage referrals Monday to Friday, from 9 am to 5 pm. All Hospital Eye Service (HES) clinics are linked to e-RS and appointments are booked electronically directly into HES clinics in accordance with agreed triaging guidelines.

#### Two level urgent ophthalmic services

All adults & children with serious conditions requiring hospital eye care within 2 weeks are referred to the HES rapid access clinic (RAC) via a standardized referral form (Supplementary Document 2) through a secure nhs.net email account based on agreed referral criteria. Referrals are triaged by RAC clinicians twice daily and patients are given specific appointments to RAC clinics or directed to MECS, if pathology is deemed non-severe post triaging. For all other non-sight or life-threatening urgent conditions, patients are seen in the community by MECS, either via e-RS, RAC redirected referrals or as walk-ins.

**Figure 1:**
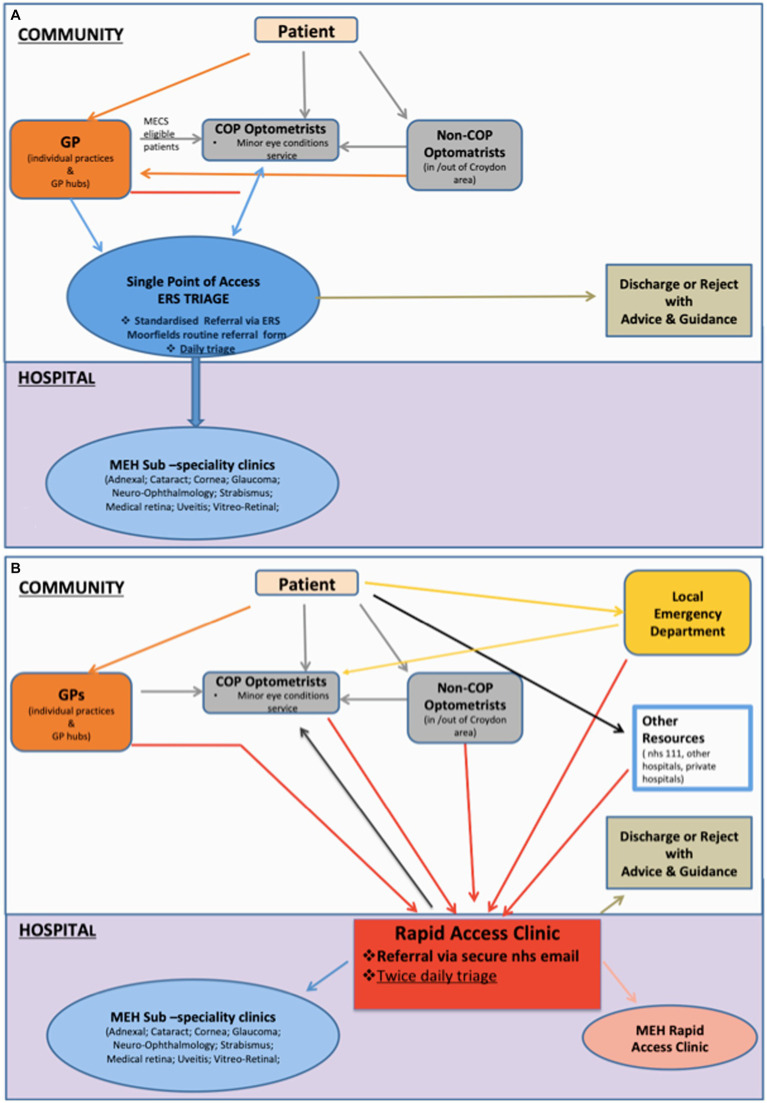
Routine **(A)** and acute **(B)** referral pathways.

The routine and urgent referral pathways are summarized in Figure 1.

#### Cataract high value pathways

Cataract and cataract-related laser referral refinement, and postoperative cataract service, follow Royal College of Ophthalmologists and Southwest London commissioning guidance (9) supplemented with a standard operating process (SOP), which involves a specified level of additional training for optometrists participating in community postoperative pathways.

#### Glaucoma high value pathways

Community optometrists provide National Institute for Health and Care Excellence (NICE) - compliant level II (repeat readings and enhanced case finding) glaucoma referral filtering care, with a pathway for level III care (referral refinement) to be mobilized within the first year of the new contract (10). The content of each pathway and level of optometrist qualifications are delineated by NICE guidelines but supplemented with a detailed agreed SOP involving local governance elements. In the UK, NICE provides national guidance and advice to improve health and social care (10).

### Communications and raising awareness of the new service

Deciding the right communication strategy (Supplementary Table 1) was key to success. Responsibilities were clearly defined: MEH had oversight of all communications among parties participating in the contract; the CCG led communications with GPs, COP led communications with other optometric practices.

The COP and MEH have ongoing activities to raise awareness, with 2 to 3 educational events per year for GPs and optometrists, which include discussions on the relevant pathways, key information required in referrals and educational content to maximize service effectiveness. Representatives from COP and MEH also attended the 6 GP networks or GP open days on the launch of the service, and provide updates / service overviews to GPs as necessary (minimum annually).

### Governance

The Care Quality Commission (CQC) in the UK regulates hospital eye services but does not cover optical practices (11). Hence, the Lead Provider model carried the burden of creating a robust governance model, which would safeguard patient safety, the reputation of the Lead Provider and the ability of the COP to deliver high quality care.

During the contract design and mobilization phase, regular meetings took place with all 3 parties (MEH, COP, CCG) covering governance, performance and contracts, with actions as required. The COP would be accountable for all community aspects of clinical governance through an effective system of quality and risk management, in line with the requirements of Standards for Better Health (12), which was designed by MEH.

Key elements of the governance framework were as follows.

#### Patient safety

The COP worked with all participating optometrists to be able to identify incidents and near misses. Incidents have to be reported weekly to the MEH risk management team and are added to the MEH incident reporting system. These have to be investigated within 28 days by the COP. Any incident of moderate or above harm or an incident that has the potential to be declared as a Serious Incident (SI) is discussed at the weekly MEH SI Reporting & Management Group with attendance of a COP representative. Incidents graded as moderate or above require a Duty of Candor letter of apology, which is written to the patient, for which the content is agreed between the MEH risk and clinical teams and COP. The COP receives a weekly email report of all reported and open incidents for monitoring purposes and has to provide assurance at the monthly MEH Quality Forum that incidents are being reported and investigated in a timely manner. The same applies to Central Alerting System notifications.

#### Patient experience

The COP is obliged to have Patient Advice Liaison Service (PALS) information available to patients. All formal or informal patient complaints, as well as PALS enquiries to the COP, have to be reported to the MEH quality team within 24 h of receipt. Monitoring actions given to staff during or following a complaint investigation are managed via the same process as incident management.

Friends and family tests (FFT) are also mandatory in all community practices. The COP provides updates at the monthly MEH Quality Forum of FFT responses and feedback from patients, themes are identified and actions are assigned.

#### Clinical effectiveness and innovation

The COP supports audits by recording patient-level information on how patients access the service, details of any appointments completed and the outcomes of those appointments. Audits are completed in line with an agreed process with joint representation from MEH and the COP. Examples of quarterly audits involve appropriateness of referral/triage decisions, adherence with protocols/defined pathways and quality of record keeping. All relevant audits are recorded on the MEH audit management software (Safeguard). The COP receives weekly email reports of all open audits for monitoring purposes and has to provide assurance at the monthly MEH Quality Forum that audits are progressing in a timely manner, and present reports for completed audits with an action plan.

The exact type of and frequency of audits to be completed is delineated within the contract (see all KPIs in Supplementary Table 1).

#### Risk registers

The COP maintains a risk register of all risks that may impact on care. These risks are discussed by the divisional management team during the monthly risk register review and transferred to the MEH South divisional risk register as required. The COP receives a weekly email report of all open actions relating to risks for monitoring purposes. The COP has been providing assurance at the monthly MEH quality forum that risks are reviewed and escalated/de-escalated in a timely manner and actions assigned are completed as necessary.

## Discussion

The Croydon model of integrated care in ophthalmology was generated as a response to financial and patient care challenges and represented a large healthcare transformation initiative. We are not aware of any similar example of such a comprehensive integrated eye service and there were few existing references to assist the design and materialization of the scheme. The venture was complex, with unexpected challenges arising and such radical system transformation required significant time commitment, inclusiveness, shared narrative and strong leadership.

There are some specific lessons that have been learnt from this journey.

Having only two main providers (one hospital, one community) within the borough was a major facilitator. Other areas in the country with multiple providers might find undertaking the process more complex and time-consuming. Also, a stable optometry sector in the locality is extremely helpful to ensure relationships are maintained and develop over time, there is buy-in for the longer term, the investment in professional development is warranted and the expertise and experience of optometrists with bespoke development, who are used to working in an integrated system delivering enhanced care is retained. However, similar integration projects will still be possible, provisionally the Lead Provider orchestrates a robust procurement process for a COP.

Another important lesson learnt was that establishing integrated care is a multifaceted and long-term process, with two clear implementation phases, the ‘contract design’ phase and the ‘mobilization’ phase. It is advisable that the project is led by a formal project management team for adequate oversight of all phases and adherence to timelines. Long-term viability and success of the integration will not be possible if attention is not paid equally to both phases. The ‘contract’ phase is when the groundwork is done, so that the contract will provide the appropriate financial envelope, corresponding to accurate activity assumptions and a realistic redesign of patient pathways. This requires good confidence in both the level of activity and assumptions about growth and estimated efficiencies. The second phase defines the governance elements, which will safeguard the quality of care. It is much easier if the trust has robust audit data relating to patient diagnoses, outcomes of hospital visits, quality of referrals, discharge rates etc to supplement national quality recommendations. This phase therefore needs to involve very close collaboration with the trust quality team. As community optometry schemes are not subject to CQC scrutiny, it is of upmost importance that the governance framework is very detailed and supported by the trust’s process.

During both phases of integration, aligning bottom-up and top-down integrators is also crucial to success, as it creates the shared narrative. The top-down intervention in our paradigm followed the traditional formal planning approach, including resource allocation and design of the wider picture of a new model of care. The shared leadership/bottom up approach was a self-discovery journey, where all stakeholders were invited to actively participate in the design of the constituents of the new model but always in line with national guidance (13).

Our approach has created a system that is scalable and can be replicated in other settings, whilst continuity will not be hindered by future leadership changes within the CCG, MEH or the community.

It is worth noting that, among the many challenges of the integrated care journey, the most challenging were the ones related to information technology (IT) and connectivity. A few examples of such encounters involve getting community optometrists to work on a platform that would connect to e-RS, granting access to e-RS in the community and linking the trust’s clinics to e-RS accurately. We would strongly advise having a dedicated IT lead within the trust, who would have a close oversight of all IT related pathways. Appropriate remuneration for IT-related costs in the community (e.g., an e-RS compatible platform which can also ideally integrate with optometry patient management systems) should also be included in the contract.

A summary of lessons learned during the mobilization and implementation phases of our contract can be found in Table 1.

**Table 1:** Lessons learned from the implementation of an integrated care contract.

Topic	Lessons
Feasibility	Using a lead provider model can overcome the numerous current barriers (e.g., lack of a national integration contract and payment structure) to allow the establishment of a fully integrated eye care service across primary, community and secondary care. Creating an integrated pathway is simpler where there are fewer main providers of community and secondary eye care and a stable primary care optometry population.
Development and leadership	Planning, implementing and delivering an integrated pathway was more complex than predicted, with many aspects to consider and under the influence of many factors. Significant time commitment is required from clinical leaders across the pathway, including commissioners, service managers and a formal project manager is crucial. The involvement of third sector and patient representation is also important.
Culture	There needs to be a vision, shared narrative and a multi-functional working culture, with collaborative relationships between clinical, commissioner and management professionals across organizational boundaries. An inclusive approach combining top-down aspects, but facilitating bottom-up innovation is central to success. An understanding that secondary care is evolving into a commissioning role, being responsible for setting high standards of care, supporting and nurturing innovative ideas.
Quality, safety and governance	There needs to be shared accountability of all participating parties. All clinical professionals in the pathway will be subject to their regulatory body requirements, but community optometry is not subject to scrutiny by national regulatory bodies. However, patients flow across organizational and sector boundaries. Therefore, the governance framework needs to be very detailed and supported by the hospital processes, providing the opportunity for shared learning, reflection and be able to follow up agreed key performance indicators (KPIs) alongside action plans for improvement. More specifically, provisions must be put in place to modernize and align secondary and primary care quality processes and systems on patient feedback collection, risk register management, incident reporting and complaint management. Provisions should be considered to reduce to absolute minimum digital exclusion, and ensure accessibility of services provided for local population.
Professional development and support	It is important to provide education and support for all parties involved and supplement national educational qualifications with local development and support specific to the local pathway and environment, especially for primary care eye teams.
Information and Technology (IT)	The most challenging aspect is IT and connectivity. It is important to invest in IT systems and staff with digital expertise.
Phases	Establishing an integrated pathway is a long-term process. There are two implementation phases: contract design and mobilization. However, there will be further phases where feedback, learning and changing population needs will drive further development of the services. For implementation phases it is crucial to use local audit and performance data, as well as national guidance to underpin key assumptions.

The process of transformation of ophthalmic care described in this paper was undertaken in a context of severe economic limitations for healthcare and within a socially deprived borough. We have demonstrated that multidimensional system changes, such as introducing integrated care service delivery, are possible, although the interplay between context, design and implementation is more complex than expected. There are multiple benefits for patients in terms of timely access to care, care closer to home, easy navigability of the care system and care under one consistently-badged service system avoiding fragmentation of care pathways from multiple providers in the pathway.

## Data availability statement

The original contributions presented in the study are included in the article/Supplementary material, further inquiries can be directed to the corresponding author.

## Author contributions

EN, RA, KM, and ZM organized the database. EN wrote the first draft of the manuscript. EN, MH, and ZM wrote sections of the manuscript. All authors contributed to conception and design of the study, manuscript revision, read, and approved the submitted version.
